# Magnesium^☆^

**DOI:** 10.1016/j.advnut.2026.100747

**Published:** 2026-10-07

**Authors:** Taylor C Wallace, Rebecca B Costello, Andrea Rosanoff

**Affiliations:** 1 Think Healthy Group, LLC, Washington, DC, United States; 2 Gerald J. and Dorothy R. Friedman School of Nutrition Science and Policy, Tufts University, Boston, MA, United States; 3 Center for Magnesium Education and Research, Pahoa, HI, United States

**Keywords:** magnesium, magnesium deficiency, hypomagnesemia, dietary magnesium, magnesium supplementation, magnesium bioavailability, dietary reference intakes, serum magnesium, magnesium reference intervals, Magnesium Depletion Score

## Magnesium

Magnesium (Mg^2+^) is an essential mineral and the primary intracellular divalent cation. It acts as an activator or cofactor in >600 enzymatic reactions, including ATP-dependent energy transfer, oxidative phosphorylation, glycolysis, protein and nucleic acid synthesis, glucose metabolism, ion transport, antioxidant defense, and cell signaling. Mg^2+^ is required for neuromuscular transmission, normal cardiac rhythm, vascular tone, and skeletal mineralization. The adult body contains ∼25 g of Mg^2+^; 50% to 60% is in bone, most of the remainder is in muscle and other soft tissues, and <1% is extracellular. Homeostasis reflects intestinal absorption, renal filtration and reabsorption, and exchange with bone. Typically, 30% to 50% of dietary Mg^2+^ is absorbed, with fractional absorption increasing when intake is low. Because >99% of body Mg^2+^ is intracellular or in bone, serum Mg^2+^ is a practical but imperfect status marker and may remain within conventional laboratory intervals despite declining tissue stores [1], [2].

## Deficiencies

Overt Mg^2+^ deficiency caused by low intake alone is uncommon in otherwise healthy individuals because the kidneys conserve Mg^2+^. Deficiency more often reflects chronic diarrhea or malabsorption; inflammatory bowel disease or celiac disease; pancreatitis; bariatric or intestinal surgery; alcohol use disorder; poorly controlled diabetes; inherited or acquired renal tubular losses; or medications that increase gastrointestinal or urinary losses. Common medication-related causes include long-term proton pump inhibitor use, loop or thiazide diuretics, aminoglycosides, cisplatin, calcineurin inhibitors, and selected epidermal growth factor receptor-targeted therapies. Older adults are especially vulnerable due to reduced intake and absorption, increased urinary losses, multimorbidity, and polypharmacy [1], [2].

Early manifestations are nonspecific and may include reduced appetite, nausea, fatigue, and weakness. Progressive depletion may cause paresthesia, tremor, muscle cramps, carpopedal spasm or tetany, seizures, delirium, and ventricular arrhythmias; refractory hypokalemia and hypocalcemia can coexist. A serum concentration below 0.75 mmol/L (1.82 mg/dL) is commonly used to define hypomagnesemia, although laboratory cutoffs vary, and concentrations below ∼0.50 mmol/L are generally associated with more clinically apparent symptoms. Chronic latent Mg^2+^ deficiency (CLMD) describes depleted tissue stores despite a serum value remaining within the conventional reference interval; concentrations ≥0.85 mmol/L are generally considered adequate to prevent this condition [3]. Assessment should therefore integrate dietary intake, symptoms, medications, kidney function, and serum Mg^2+^, including the serum Mg^2+^-to-calcium ratio, and, when useful, urinary Mg^2+^ rather than relying on a single test [1], [2], [4], [5].

## Diet Recommendations

The United States and Canadian Dietary Reference Intakes for Mg^2+^ have remained unchanged since 1997; no updated United States/Canadian values have been adopted by the National Academy of Medicine as of October 2026 [4], [6]. The current Recommended Dietary Allowances (RDAs) and infant Adequate Intakes (AIs) are shown in Table 1.

**TABLE 1:** United States and Canadian Dietary Reference Intakes for magnesium

Life stage	Male, mg/d	Female, mg/d	Pregnancy, mg/d	Lactation, mg/d
0–6 mo	30^1^	30^1^	—	—
7–12 mo	75^1^	75^1^	—	—
1–3 y	80	80	—	—
4–8 y	130	130	—	—
9–13 y	240	240	—	—
14–18 y	410	360	400	360
19–30 y	400	310	350	310
31–50 y	420	320	360	320
≥51 y	420	320	—	—

All other values are Recommended Dietary Allowances. Values represent total intake from food, beverages, and supplements [6].

1 Adequate intake.

A recent international development is the Nordic Nutrition Recommendations 2023, which established adult AIs of 350 mg/d for men and 300 mg/d for women, with provisional average requirements of 280 and 240 mg/d, respectively. These values were based on observed intakes and available balance evidence because no adequate functional biomarker or new balance studies permitted a more definitive requirement estimate [7]. Contemporary United States data continue to show substantial shortfalls. In What We Eat in America (NHANES August 2021–August 2023), the mean daily Mg^2+^ intake from foods and beverages was 330 mg/d in men aged ≥20 y and 264 mg/d in women; among adolescents aged 12 to 19 y, the corresponding means were 295 and 218 mg/d, respectively [8]. Usual intake modeling from NHANES 2017 through March 2020 estimated that 57% of adult men, 52% of adult women, 81% of boys aged 14 to 18 y, and 89% of girls aged 14 to 18 y consumed less Mg^2+^ than the Estimated Average Requirement (EAR) from foods and beverages [9]. When intake from dietary supplements was included, the estimated prevalence of Mg^2+^ intake below the EAR decreased to 51% among adult men and 45% among adult women, indicating that supplements reduce but do not eliminate population-level inadequacy [10].

## Food Sources

Mg^2+^ is widely distributed in foods, especially green leafy vegetables, legumes, nuts, seeds, whole grains, soy foods, cocoa, and selected fortified cereals. Fish, dairy foods, meats, fruits, and vegetables also contribute to Mg^2+^ intake because they are commonly consumed. Examples include roasted pumpkin seeds (156 mg/oz), chia seeds (111 mg/oz), almonds (80 mg/oz), cooked spinach (78 mg/½ cup), and cooked black beans (60 mg/½ cup). Refining grains removes the Mg^2+^-rich bran and germ, whereas mineral and tap waters can provide meaningful amounts of Mg^2+^, depending on the source; bottled water can contain from negligible to >120 mg/L. Dietary patterns that emphasize minimally processed plant foods generally provide more Mg^2+^ than those dominated by refined grains and highly processed foods [4]. In 2022, the United States Food and Drug Administration announced that it would exercise enforcement discretion for the following qualified health claim for products that provide ≥84 mg of Mg^2+^ per the Reference Amount Customarily Consumed (RACC): “*Consuming diets with adequate magnesium may reduce the risk of high blood pressure (hypertension). However, the FDA has concluded that the evidence is inconsistent and inconclusive.*” Supplements bearing the claim should provide no more than 350 mg/d when used as labeled, and conventional foods must meet the “low sodium” criterion and other applicable requirements [11].

## Clinical Uses

Mg^2+^ is used clinically to correct documented deficiency. Oral replacement is generally appropriate for mild or asymptomatic depletion, whereas intravenous Mg^2+^ is used for severe or symptomatic hypomagnesemia, marked arrhythmias or seizure risk, or inability to absorb or tolerate oral therapy. Dosing and monitoring must account for kidney function. Intravenous or intramuscular Mg^2+^ sulfate remains the anticonvulsant of choice for preventing and treating eclamptic seizures and is also used for fetal neuroprotection before anticipated early preterm birth. Intravenous Mg^2+^ is used to treat torsades de pointes and selected polymorphic ventricular tachyarrhythmias associated with QT prolongation. Mg^2+^ hydroxide, oxide, citrate, and sulfate are also used in antacid and osmotic laxative products [1], [2], [4], [12].

### Supplement form and bioavailability

The Supplement Facts Panel reports elemental Mg^2+^ rather than the total mass of the Mg^2+^ salt. Absorption depends largely on solubility: citrate, chloride, lactate, and aspartate generally dissolve more readily and are absorbed more completely than oxide or sulfate. Mg^2+^ oxide provides a high elemental Mg^2+^ fraction by weight but has low fractional absorption and greater laxative potential. Glycinate (bisglycinate) and malate are often selected for perceived gastrointestinal tolerability, but direct head-to-head human evidence showing superior absorption remains somewhat limited. Mg^2+^ oxalate is uncommon as a commercial supplement form; oxalate is more often relevant as a dietary ligand that can complex divalent minerals. Form selection should consider elemental dose, renal function, gastrointestinal tolerance, intended use, and product quality rather than the salt name alone [2], [4], [6].

## Toxicity

No Tolerable Upper Intake Level (UL) has been established for Mg^2+^ naturally present in foods and beverages. The United States and Canadian UL applies only to Mg^2+^ from dietary supplements and medications: 65 mg/d for ages 1 to 3 y, 110 mg/d for ages 4 to 8 y, and 350 mg/d from age 9 y through adulthood, including pregnancy and lactation [6]. The critical adverse effect used to derive the UL is osmotic diarrhea; nausea and abdominal cramping can also occur. A 2023 perspective called for re-evaluation of the adult supplemental UL because the evidence base is outdated, heterogeneous, and focused on mild gastrointestinal effects, but no official United States or Canadian revision has been adopted [13].

Clinically important hypermagnesemia is uncommon in people with normal kidney function but can occur with renal impairment, bowel obstruction or dysmotility, large doses of Mg^2+^-containing laxatives or antacids, or excessive parenteral administration. Progressive toxicity can cause flushing, nausea, hypotension, lethargy, diminished deep-tendon reflexes, muscle weakness, respiratory depression, conduction abnormalities, and cardiac arrest. Mg^2+^ can reduce the absorption of oral bisphosphonates and tetracycline or quinolone antibiotics; product labeling should be used to separate administration times. Drugs such as proton pump inhibitors and diuretics can also alter Mg^2+^ status and warrant monitoring during prolonged use [1], [2], [4].

## Recent Research

### Magnesium Depletion Score

The Magnesium Depletion Score (MDS) is a noninvasive composite score intended to identify factors that impair Mg^2+^ absorption or increase Mg^2+^ loss. It was derived from Mg^2+^-retention testing and later applied to United States NHANES cohorts. Higher scores indicate greater risk of depletion [14]. The MDS assigns 1 point each for current diuretic use, current proton pump inhibitor use, heavy alcohol use (>1 drink/d for women or >2 drinks/d for men), and an estimated glomerular filtration rate (eGFR) of 60 to <90 mL/min/1.73 m^2^. An eGFR < 60 mL/min/1.73 m^2^ is assigned 2 points. A score >2 has frequently been used to indicate an elevated risk of Mg^2+^ depletion, although cut points vary by population and outcome [14]. A recent 2025 scoping review [14] included 48 articles: 39 cross-sectional analyses, 15 prospective cohort analyses, and 1 secondary analysis of a randomized trial; some articles contributed more than 1 analysis. All but 2 analyses reported adverse associations with a higher MDS, and dietary Mg^2+^ intake was inversely related to the score. Reported outcomes included systemic inflammation, cardiovascular disease, all-cause mortality, hypertension, diabetes, chronic kidney disease, osteoporosis, frailty, and several other chronic conditions. The MDS is a promising, rapid, low-cost adjunct to dietary assessment, but it is not yet a stand-alone diagnostic test. The evidence is dominated by adult observational NHANES analyses; scoring practices and cut points vary; kidney function contributes heavily to the score; and clinical trials of MDS-guided interventions are needed [14].

### Contemporary serum Mg^2+^ reference intervals

NHANES 2021 to 2023 measured serum Mg^2+^ in a nationally representative United States sample for the first time since NHANES I (1971–1974). The analysis included 787 adolescents aged 12 to 18 y and 5474 adults aged ≥19 y, and estimated sex-specific reference intervals using International Federation of Clinical Chemistry procedures (Table 2). Mean concentrations were lower in adults with diabetes, hypertension, or chronic kidney disease than in metabolically healthy adults, though the differences were small. Using the proposed CLMD threshold of <0.85 mmol/L (∼2.06–2.07 mg/dL), 67.8% of United States adults were classified as at risk. This estimate should not be interpreted as the prevalence of clinically proven deficiency; the threshold is not universally accepted, serum Mg^2+^ is an imperfect proxy for tissue stores, and the new intervals are population-based rather than outcome-optimized treatment targets. Nevertheless, the findings support incorporating serum Mg^2+^ into clinical evaluation when depletion risk is present and interpreting it alongside dietary intake, medications, kidney function, urinary Mg^2+^, and potentially the MDS [3], [15].

**TABLE 2:** Contemporary United States population reference intervals for serum magnesium

Population	mg/dL	mmol/L	mEq/L
Boys, 12–18 y (total population)	1.70–2.19	0.70–0.90	1.40–1.80
Girls, 12–18 y (total population)	1.64–2.18	0.68–0.90	1.35–1.79
Men, ≥19 y (metabolically healthy)	1.72–2.26	0.71–0.93	1.42–1.86
Women, ≥19 y (metabolically healthy)	1.70–2.21	0.70–0.91	1.40–1.82

Intervals are the 2.5th to 97.5th percentiles [15].

## For Further Information

For comprehensive reviews and assessment guidance, see de Baaij et al. [1], the NIH Office of Dietary Supplements’ health professional fact sheet [4], and Costello and Rosanoff’s [2] magnesium chapter in Present Knowledge in Nutrition.

## Author contributions

The authors’ responsibilities were as follows—TCW: writing—original draft; TCW, RBC, and AR: conceptualization, writing—review and editing. All authors have read and approved the final manuscript submission.

## Declaration of generative AI and AI-assisted technologies in the writing process

During the preparation of this work, the authors used GPT-6 Astra (OpenAI) to assist in the gramatical editing and formatting of the manuscript. After using this tool, the authors reviewed and edited the content as needed and take full responsibility for the content of the publication.

## Funding

The authors reported no funding received for this study.

## Conflict of interest

RBC is a Senior Research Fellow of the Center for Magnesium Education and Research. AR is the Director of the Center for Magnesium Education and Research. TCW has received competitive unrestricted research grants unrelated to this work from New Capstone, Inc. TCW receives royalties from the Academy of Nutrition and Dietetics for editing the Health Professional's Guide to Dietary Supplements. TCW is on the science advisory boards of Forbes Health (unpaid), Haleon, and Deerland Probiotics & Enzymes, and is a member of the National Academy of Medicine Expert Panel on Validating Health Information Using GenAI: A Nutrition Case Study (unpaid). TCW is the Editor-in-Chief of the Journal of Dietary Supplements, Deputy Editor-in-Chief of the Journal of the American Nutrition Association, and Nutrition Section Editor of Annals of Medicine. TCW is a Senior Fellow of the Center for Magnesium Education and Research (unpaid). TCW has an up-to-date International Committee of Medical Journal Editors disclosure of interest statement available at http://www.drtaylorwallace.com.
